# Supplementary material for: Inhibition of mitochondrial folate metabolism drives differentiation through mTORC1 mediated purine sensing
Source: Nat Commun. 2024 Mar 2;15:1931. doi: 10.1038/s41467-024-46114-0 (PMC10908830; doi:10.1038/s41467-024-46114-0)
Supplement: Supplementary file 1 — Supplementary_Information [file 41467_2024_46114_MOESM1_ESM.pdf]

Inhibition of mitochondrial folate metabolism drives differentiation through mTORC1-mediated purine sensing

Martha M. Zarou[1], Kevin M. Rattigan[1], Daniele Sarnello[1], Engy Shokry[2], Amy Dawson[1], Angela Ianniciello[1], Karen Dunn[3], Mhairi Copland[3], David Sumpton[2], Alexei Vazquez[1], G. Vignir Helgason[1]

[1]Wolfson Wohl Cancer Research Centre, School of Cancer Sciences, University of Glasgow, Glasgow, G61 1QH, UK.

[2] Cancer Research UK Scotland Institute, Glasgow G61 1BD, UK.

[3] Paul O'Gorman Leukaemia Research Centre, School of Cancer Sciences, University of Glasgow, Glasgow, G12 0ZD, UK.

Corresponding Authors: Alexei Vazquez (avazque1@protonmail.com) and G. Vignir Helgason (vignir.helgason@glasgow.ac.uk)

Supplementary Information File containing:

Supplementary Figures 1-8 (and Figure Legends)

Source Data (in separate files)

Supplementary Tables 1-3

Full Western Blot membranes (uncropped and unprocessed scans of the western blots are shown in the Source Data File)

Supplementary Figure 1:

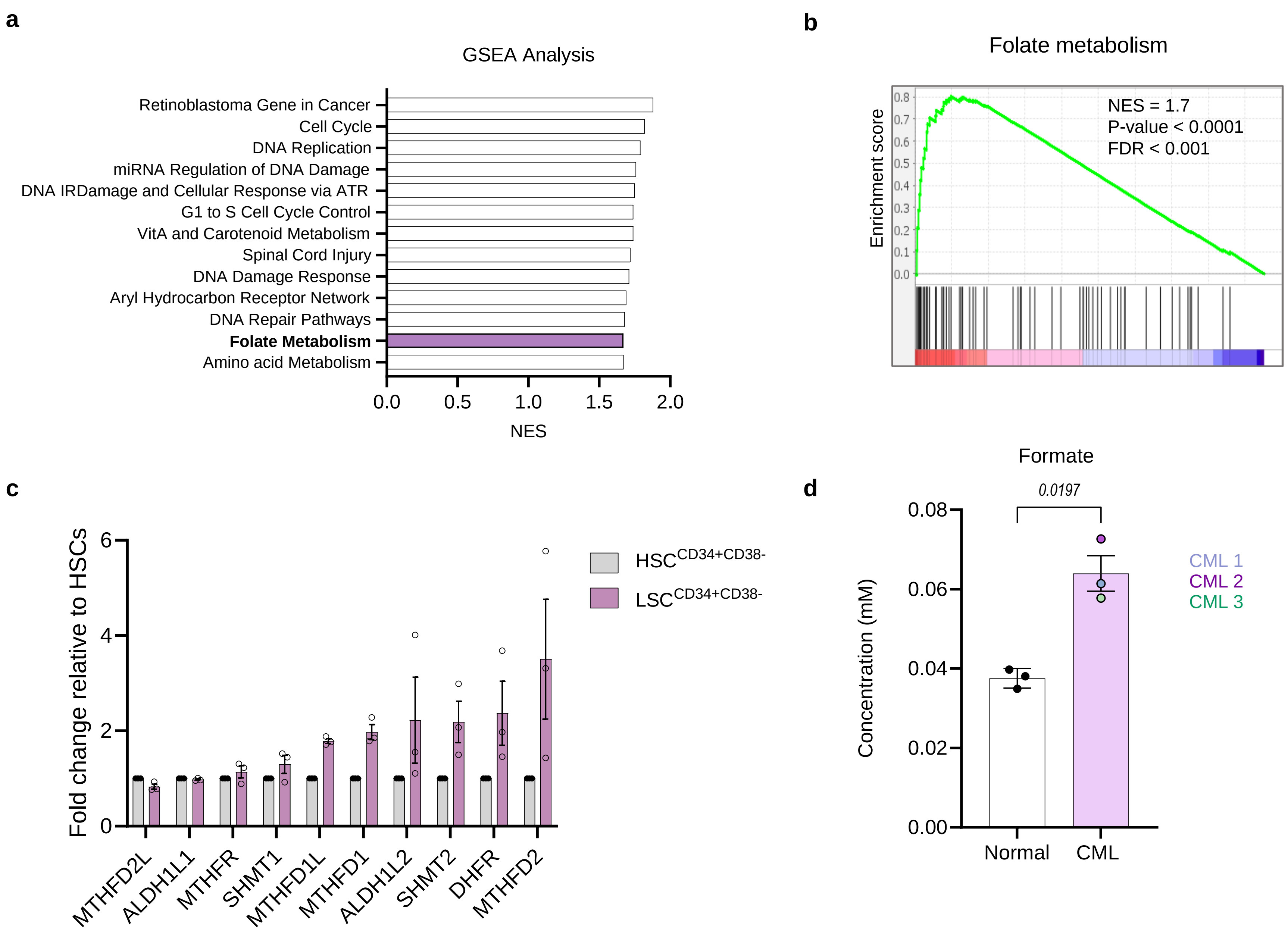

Supplementary Fig.1: Folate metabolism is deregulated in LSCs. a,b, Gene set enrichment analysis of significantly deregulated genes in CML LSCs compared to normal HSCs ($CD34^{+}CD38^{-}$) (E-MTAB-2581). NES, normalised enrichment score; FDR, false discovery rate. **c**, Expression of folate metabolism associated gene transcripts plotted in ascending order relative to HSCs. **d**, Formate concentration in media from normal and CML $CD34^{+}$ cells following 48 h culture (n=3 patient samples). Data are shown as the mean ± s.e.m. P-values were calculated using unpaired two-tailed t-test with Welch's correction (**d**). Source data are provided as a Source Data file.

Supplementary Figure 2:

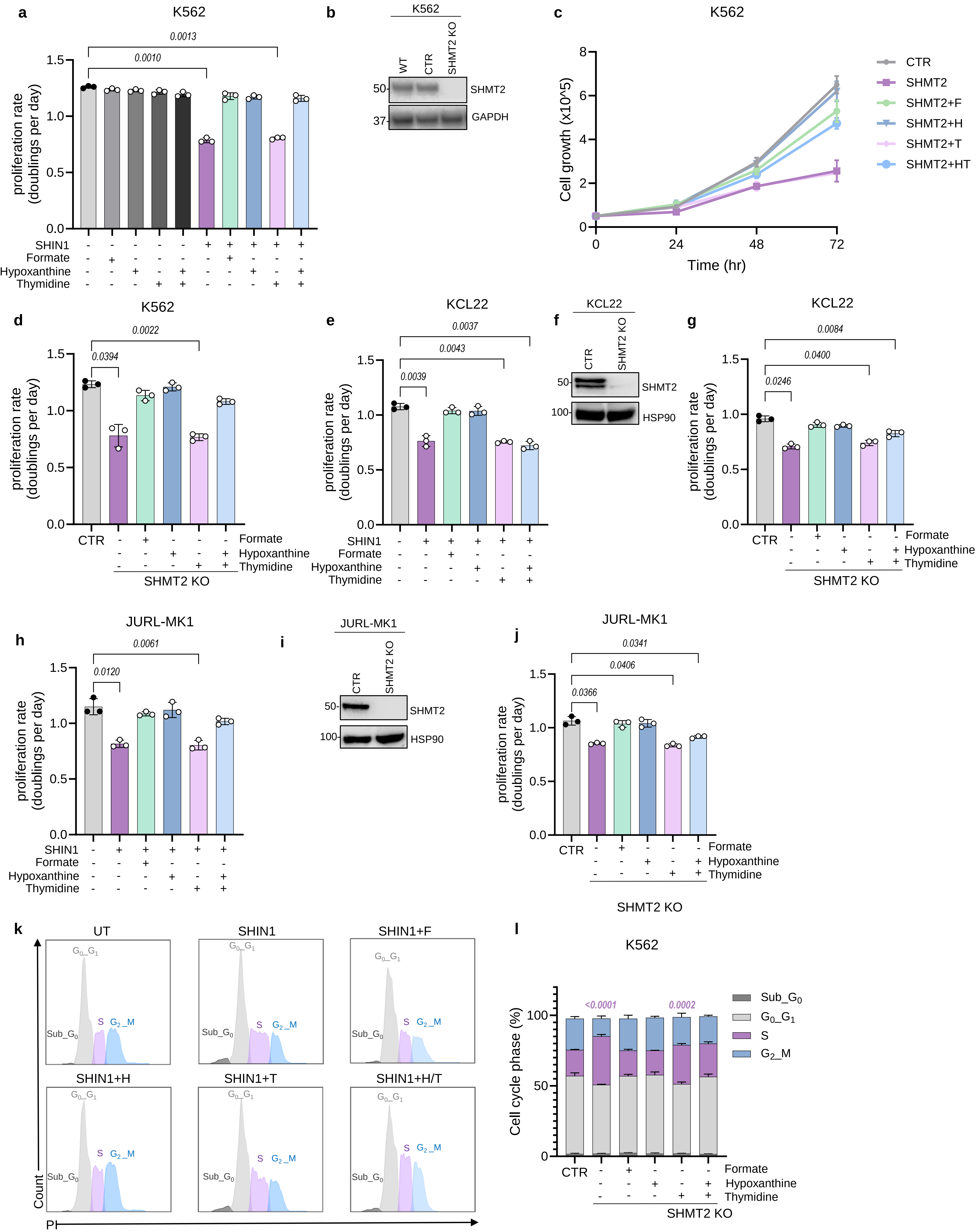

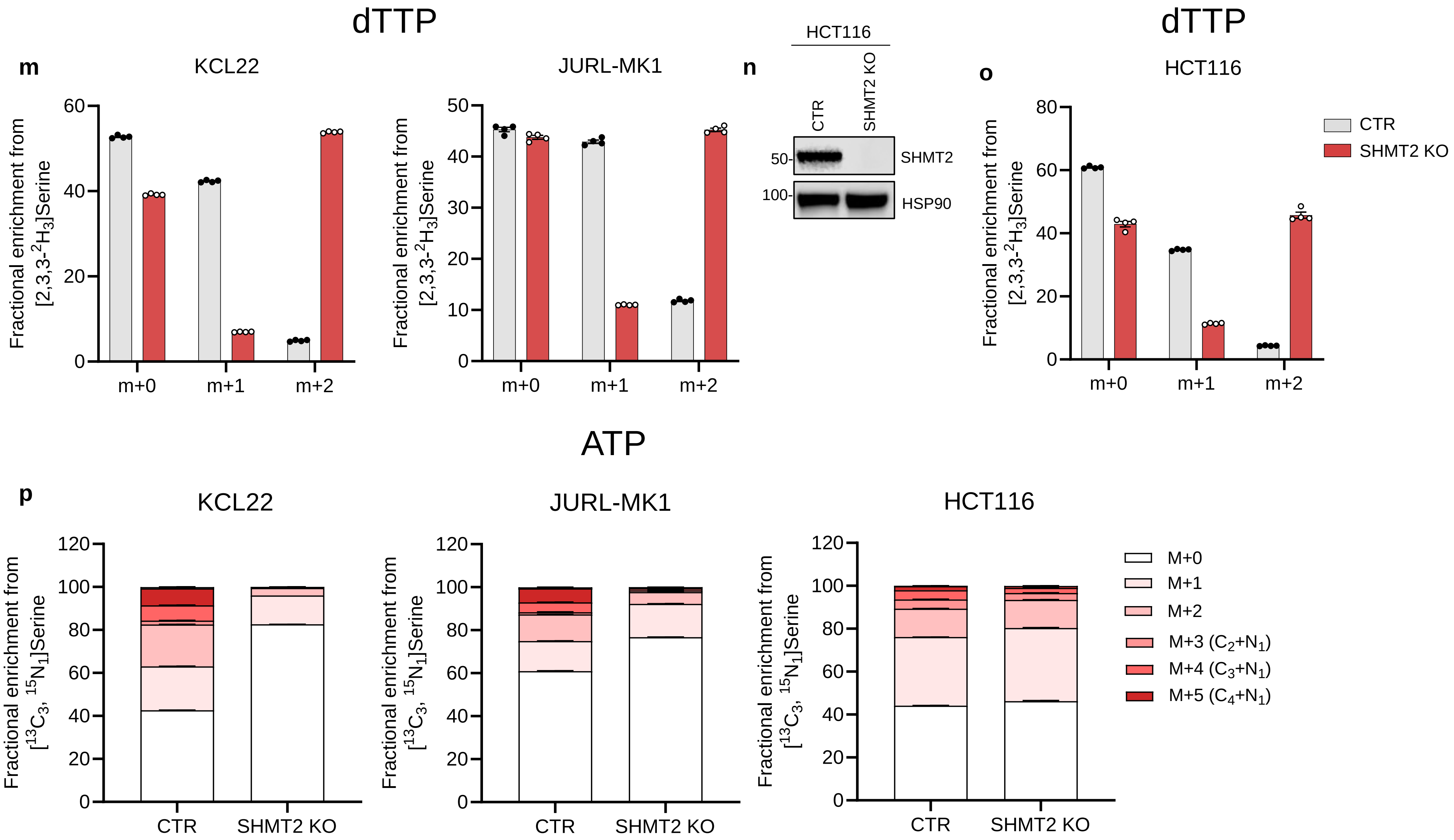

dTTP
m
KCL22
JURL-MK1
Fractional enrichment from [2,3,3-2H3]Serine
m+0
m+1
m+2
n
HCT116
CTR
SHMT2 KO
50-
100-
SHMT2
HSP90
dTTP
o
HCT116
CTR
SHMT2 KO
ATP
p
KCL22
JURL-MK1
HCT116
Fractional enrichment from [13C3, 15N1]Serine
CTR
SHMT2 KO
M+0
M+1
M+2
M+3 (C2+N1)
M+4 (C3+N1)
M+5 (C4+N1)

Supplementary Fig.2: Mitochondrial serine catabolism is required for CML cell proliferation. a, Proliferation rate of K562 cells exposed to 1 mM formate (F), 100 μM hypoxanthine (H), 16 μM thymidine (T) with or without 2.5 μM SHIN1 for 72 h (n=3 independent cultures). **b**, Immunoblot of SHMT2 levels in parental, empty vector expressing (CTR) or SHMT2 knockout (KO) K562 cells. **c,d,** Growth (**c**) and proliferation rate (**d**) of K562 CTR and SHMT2 KO cells with or without 1 mM formate, 100 μM hypoxanthine, 16 μM thymidine for 72 h (n=3 independent cultures). **e**, Proliferation rate of KCL22 cell treated as in (**a**) (n=3 independent cultures). **f**, Immunoblot of SHMT2 levels in KCL22 CTR and SHMT2 KO cells. **g**, Proliferation rate of KCL22 CTR and SHMT2 KO cells treated as in (**d**) (n=3 independent cultures).**h**, Proliferation rata of JURL-MK1 cells exposed to treatments as depicted in (**a**) (n=3 independent cultures). **i**, Western blot analysis of SHMT2 levels in JURL-MK1 CTR and SHMT2 KO cells. **j**, Proliferation rate of JURL-MK1 CTR and SHMT2 KO cells in the presence of treatments as in (**d**) (n=3 independent cultures). **k**, Histograms from cell cycle analysis of K562 cells treated as in (**a**). **l**, Percentage of cell cycle phases of K562 CTR and SHMT2 KO treated as in (**c**) (n=3 independent cultures). **m**, Mass isotopologue distribution in deoxythymidine triphosphate (dTTP) in KCL22, JURL-MK1 CTR or SHMT2 KO cells cultured with 140 μM 2,3,3-$^{2}H_3$-serine (n=4 independent wells from individual experiment). **n**, Immunoblot of SHMT2 in HCT116 CTR and SHMT2 KO cells. **o**, Mass isotopologue distribution in dTTP in HCT116 CTR or SHMT2 KO cells cultured with 140 μM 2,3,3-$^{2}H_3$-serine (n=4 independent wells from individual experiment). **p**, Fractional enrichment of ATP in KCL22, JURL-MK1, HCT116 CTR and SHMT2 KO cells cultured with 140 μM $^{13}C_3{}^{15}N_1$-serine. (n=4 independent wells from individual experiment). Data are shown as mean ± s.e.m. P-values were calculated with a repeated measure one-way ANOVA with Dunnett's multiple comparisons test (**a**,**d**,**e**,**g**,**h**,**j**) or two-way ANOVA with Tukey's multiple comparison's test (**l**). P-values in (**l**) are derived from comparing the S phase between CTR, SHMT2 KO and SHMT2 KO+T. Source data are provided as a Source Data file.

Supplementary Figure 3:

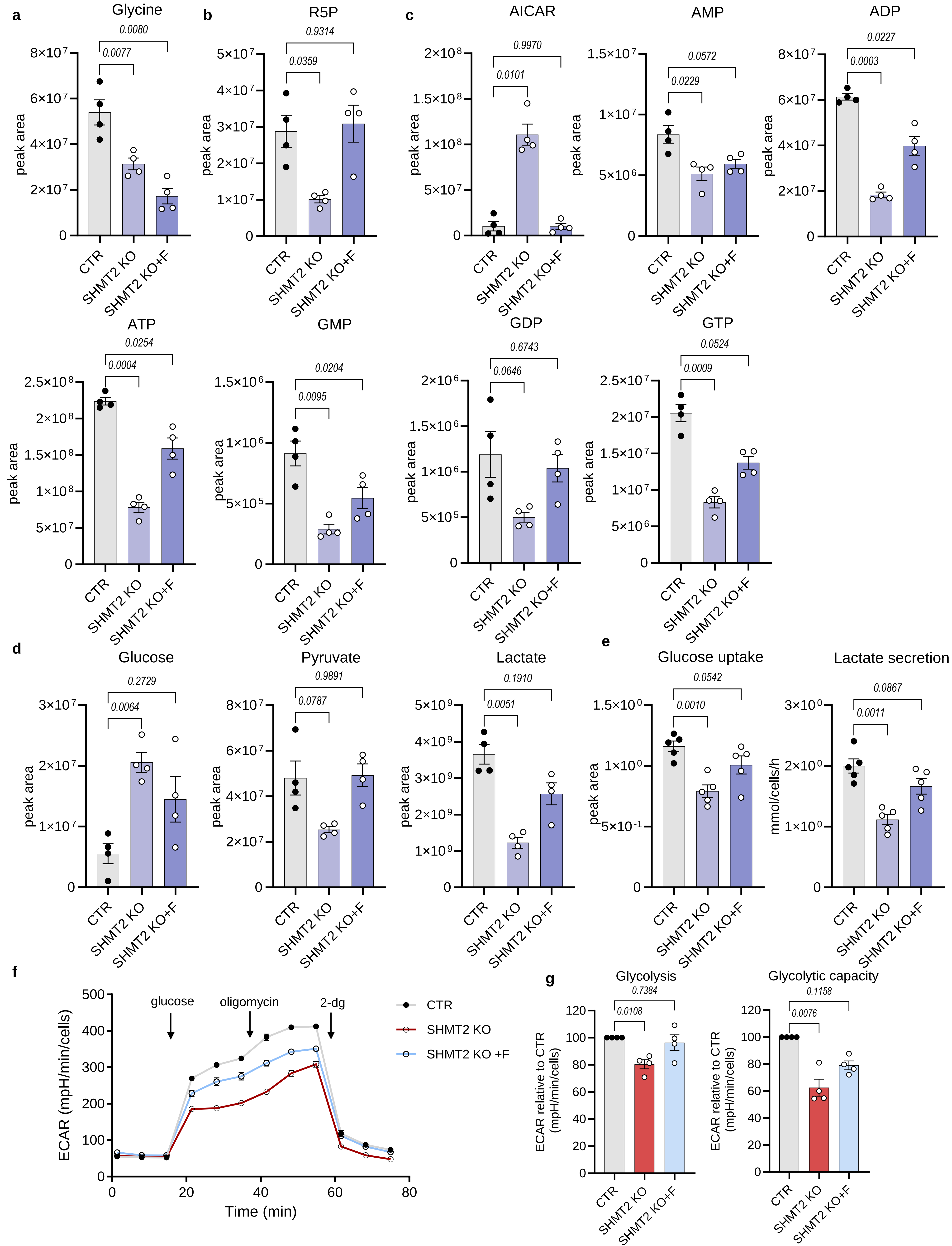

Supplementary Fig.3: Inhibition of mitochondrial serine catabolism disrupts de novo purine synthesis and glycolysis in CML cells. a-d, LC-MS measurement of intracellular glycine (**a**), ribose-5-phosphate (R5P) (**b**), purine biosynthetic intermediate and nucleotides (**c**) and glycolysis related metabolites (**d**) in K562 CTR and SHMT2 KO cells with or without the addition of 1 mM of formate for 24 h (n=4 independent cultures). **e**, Glucose uptake and lactate secretion in K562 CTR and SHMT2 KO in the presence or absence the addition of 1 mM of formate for 24 h (n=5 independent cultures). **f**,**g**, Representative extracellular acidification rate (ECAR) profile (**f**), relative glycolysis and glycolytic capacity (**g**) as measured by the Seahorse XF analyser of K562 CTR and SHMT2 KO cells cultured with or without the supplementation of 1 mM of formate (n=4 independent cultures). Data are shown as the mean ± s.e.m. P-values are derived from a repeated measure one-way ANOVA with Dunnett's multiple comparisons test (**a-e**), ordinary one-way ANOVA with Dunnett's multiple comparisons test for glycolysis and Kruskal-Wallis test with Dunn's multiple comparisons test for glycolytic capacity (**g**). Source data are provided as a Source Data file.

Supplementary Figure 4:

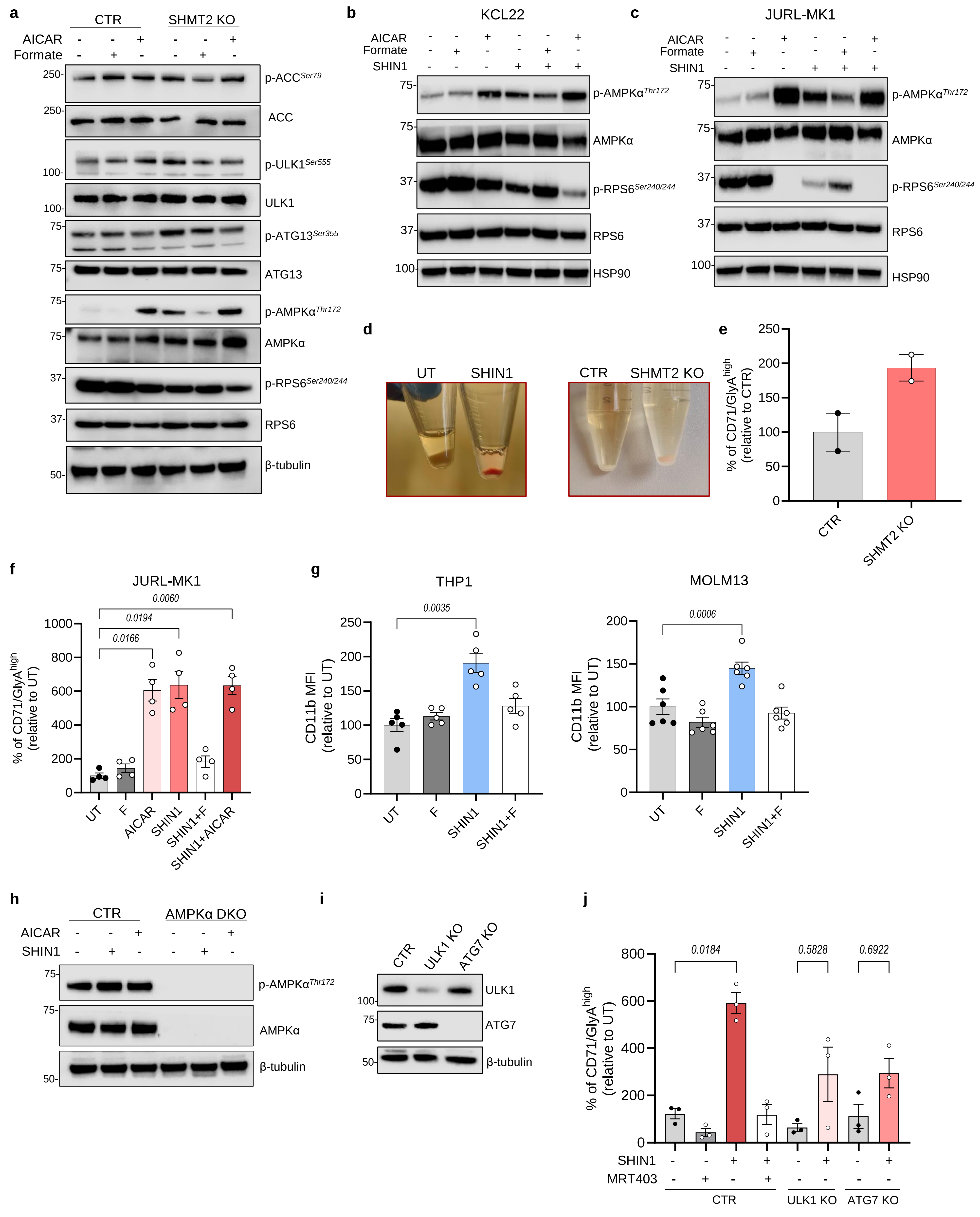

Supplementary Fig.4: AML cells are sensitive to mitochondrial 1C metabolism-induced maturation, while autophagy deficiency prevents differentiation. a, Immunoblot analysis to assess AMPK and mTORC1 signalling in K562 CTR and SHMT2 KO cells following 24 h incubation with 1mM AICAR or 1 mM formate (representative of three independent experiments). **b-c**, Immunoblot analysis to assess AMPK and mTORC1 signalling in KCL22 (**b**) and JURL-MK1 (**c**) following exposure to 1 mM AICAR, 2.5 μM SHIN1 in the presence or absence of 1 mM formate and combination of SHIN1 with AICAR for 24 h (representative of three independent experiments). **d**, Change in pellet colour of K562 cells following SHIN1 treatment or SHMT2 KO. **e**, Percentage of CD71/GlyA^{high} cells relative to CTR following loss of SHMT2 (n=2 independent cultures). **f**, Percentage of CD71/GlyA^{high} JURL-MK1 cells relative to untreated following treatment as in (**b-c**) for 72 h (n=4 independent cultures). **g**, Quantification of CD11b MFI in THP1 and MOLM13 cells relative to untreated following 72 h incubation with 2.5 μM SHIN1 with or without the supplementation of 1 mM formate (n=5 independent cultures for THP1 cells and n=6 independent cultures for MOLM13). **h**, Western blot analysis of AMPKα levels and phosphorylation in K562 CTR and AMPKα DKO cells in the presence of 1 mM AICAR or 2.5 μM SHIN1 for 24 h. **i**, Immunoblot analysis of ULK1 and ATG7 levels in K562 CTR, ULK1 KO and ATG7 KO cells. **j**, Relative percentage of CD71/GlyA^{high} K562 CTR, ULK1 KO and ATG7 KO cells treated with 2.5 μM SHIN1 for 72 h. (n=3 independent cultures). Data are presented as the mean ± s.e.m. P-values were calculated using a repeated measure one-way ANOVA with Dunnett's multiple comparisons test (**f,g,j**). Source data are provided as a Source Data file.

Supplementary Figure 5:

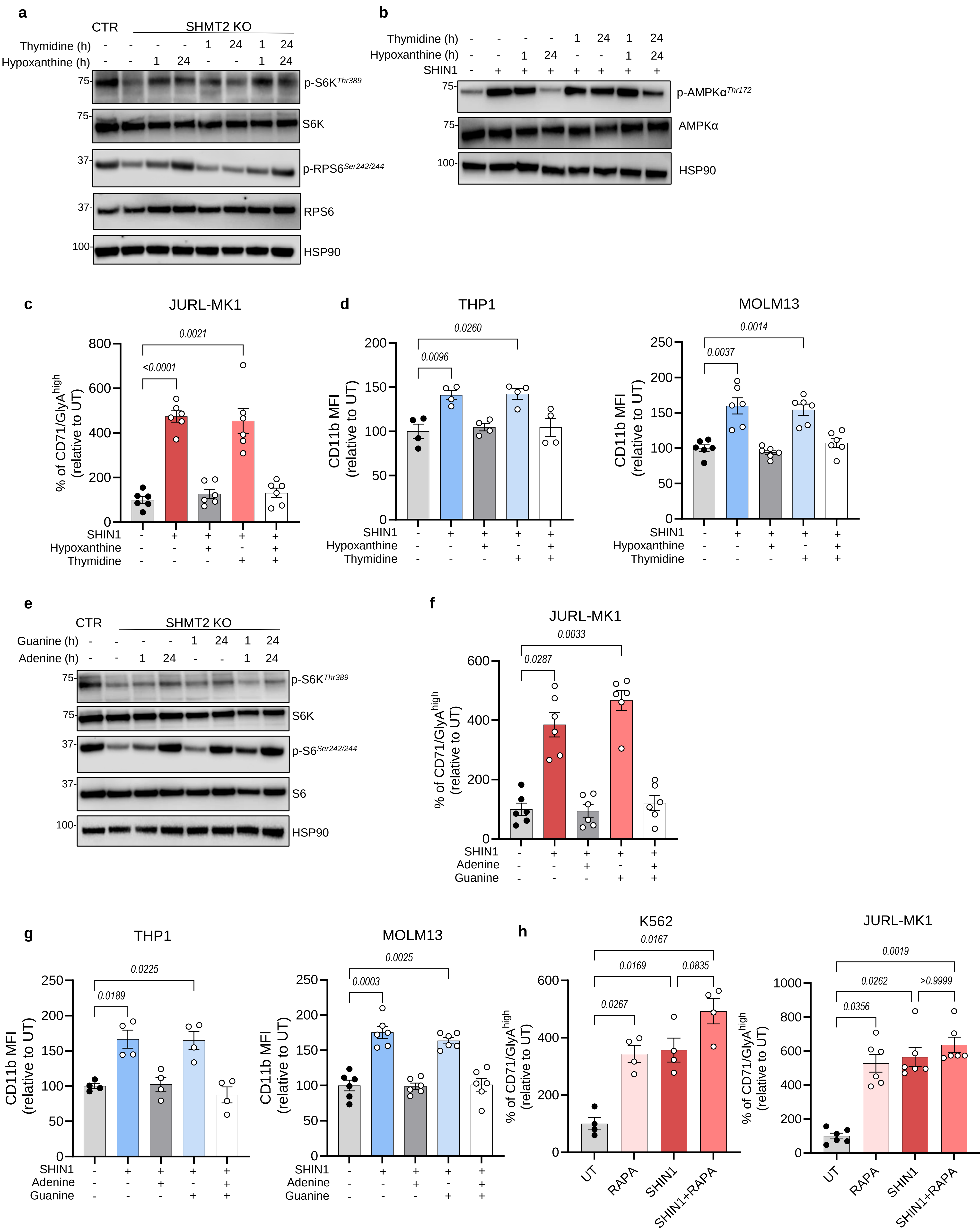

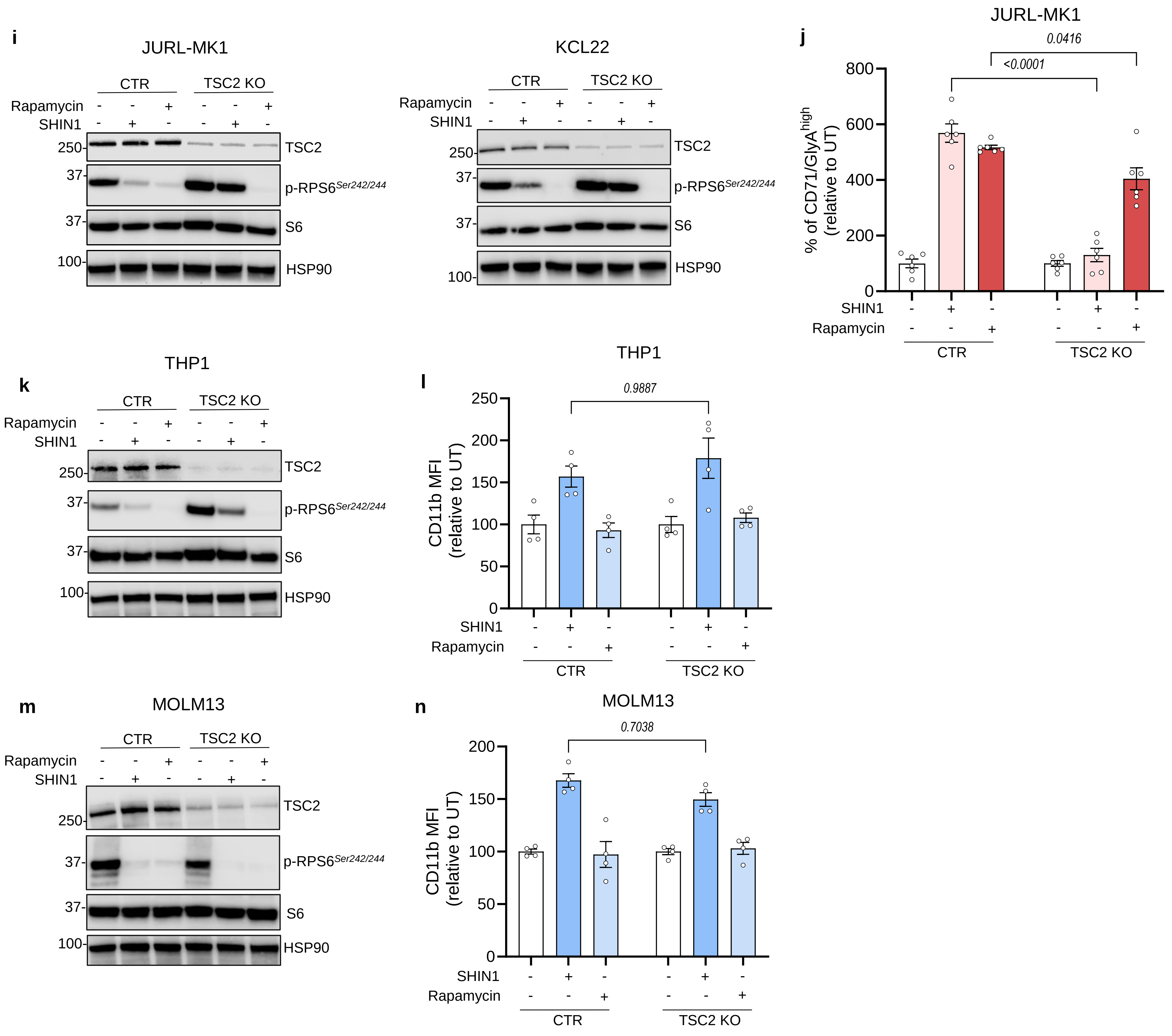
i
JURL-MK1
CTR
TSC2 KO
Rapamycin
SHIN1
250
37
37
100
TSC2
p-RPS6Ser242/244
S6
HSP90
KCL22
CTR
TSC2 KO
Rapamycin
SHIN1
250
37
37
100
TSC2
p-RPS6Ser242/244
S6
HSP90
j
JURL-MK1
0.0416
<0.0001
% of CD71/GlyAhigh (relative to UT)
800
600
400
200
0
SHIN1
Rapamycin
CTR
TSC2 KO
k
THP1
CTR
TSC2 KO
Rapamycin
SHIN1
250
37
37
100
TSC2
p-RPS6Ser242/244
S6
HSP90
l
THP1
0.9887
CD11b MFI (relative to UT)
250
200
150
100
50
0
SHIN1
Rapamycin
CTR
TSC2 KO
m
MOLM13
CTR
TSC2 KO
Rapamycin
SHIN1
250
37
37
100
TSC2
p-RPS6Ser242/244
S6
HSP90
n
MOLM13
0.7038
CD11b MFI (relative to UT)
200
150
100
50
0
SHIN1
Rapamycin
CTR
TSC2 KO

Supplementary Fig.5: Role of mTORC1 in nucleotide sensing and differentiation. a, **b**, Immunoblot of mTORC1 targets (**a**) and AMPKα phosphorylation (**b**) in K562 CTR and SHMT2 KO cells with or without 100 μM hypoxanthine and/or 16 μM thymidine for the indicated times (representative of three independent experiments). **c**, Relative percentage CD71/GlyA^{high} JURL-MK1 cells after 72 h incubation with treatments as in (**a**) (n=4 independent cultures). **d**, Quantification of CD11b MFI in THP1 and MOLM13 cells treated as in (**a**) (n=4 independent cultures for THP1 cells and n=6 independent cultures for MOLM13). **e**, Immunoblot analysis of mTORC1 targets in K562 CTR and SHMT2 KO cells with or without 30 μM adenine and/or 30 μM guanine for the indicated times (representative of three independent experiments). **f**, Relative percentage CD71/GlyA^{high} JURL-MK1 treated as in (**e**) for 72 h (n=6 independent cultures). **g**, Quantification of CD11b MFI in THP1 and MOLM13 cells treated as described in (**e**) (n=4 independent cultures for THP1 cells and n=6 independent cultures for MOLM13). **h**, Relative percentage of CD71/GlyA^{high} K562 and JURL-MK1 cells treated with 10 nM rapamycin (RAPA), 2.5 μM SHIN1, or combination for 72 h (n=4 independent cultures). **i**, Immunoblot analysis of RPS6 phosphorylation in JURL-MK1, KCL22 CTR and TSC2 KO cells incubated with 2.5 μM SHIN1 or 10nM rapamycin for 24 h (representative of three independent experiments). **j**, Relative percentage of CD71/GlyA^{high} JURL-MK1 CTR and TSC2 KO cells following treatment as in (**i**) (n=6 independent cultures). **k**, Immunoblot of RPS6 phosphorylation in THP1 CTR and TSC2 KO treated as in (**i**) for 24 h (representative of three independent experiments). **l**, Quantification of CD11b MFI in THP1 CTR and TSC2 KO cells treated as in (**i**) (n=4 independent cultures). **m**, Immunoblot of RPS6 phosphorylation in THP1 CTR and TSC2 KO cells treated as in (**i**) for 24 h (representative of three independent experiments). **n**, Quantification of CD11b MFI in MOLM13 CTR and TSC2 KO cells treated as in (**i**) (n=4 independent cultures). Data are presented as the mean ± s.e.m. P-values were calculated using a repeated measure one-way ANOVA with Dunnett's multiple comparisons test (**c**,**d**,**f**,**g**,**h**) and repeated two-way ANOVA with Sidak's multiple comparisons test (**j,l,n**). Source data are provided as a Source Data file.

Supplementary Figure 6:

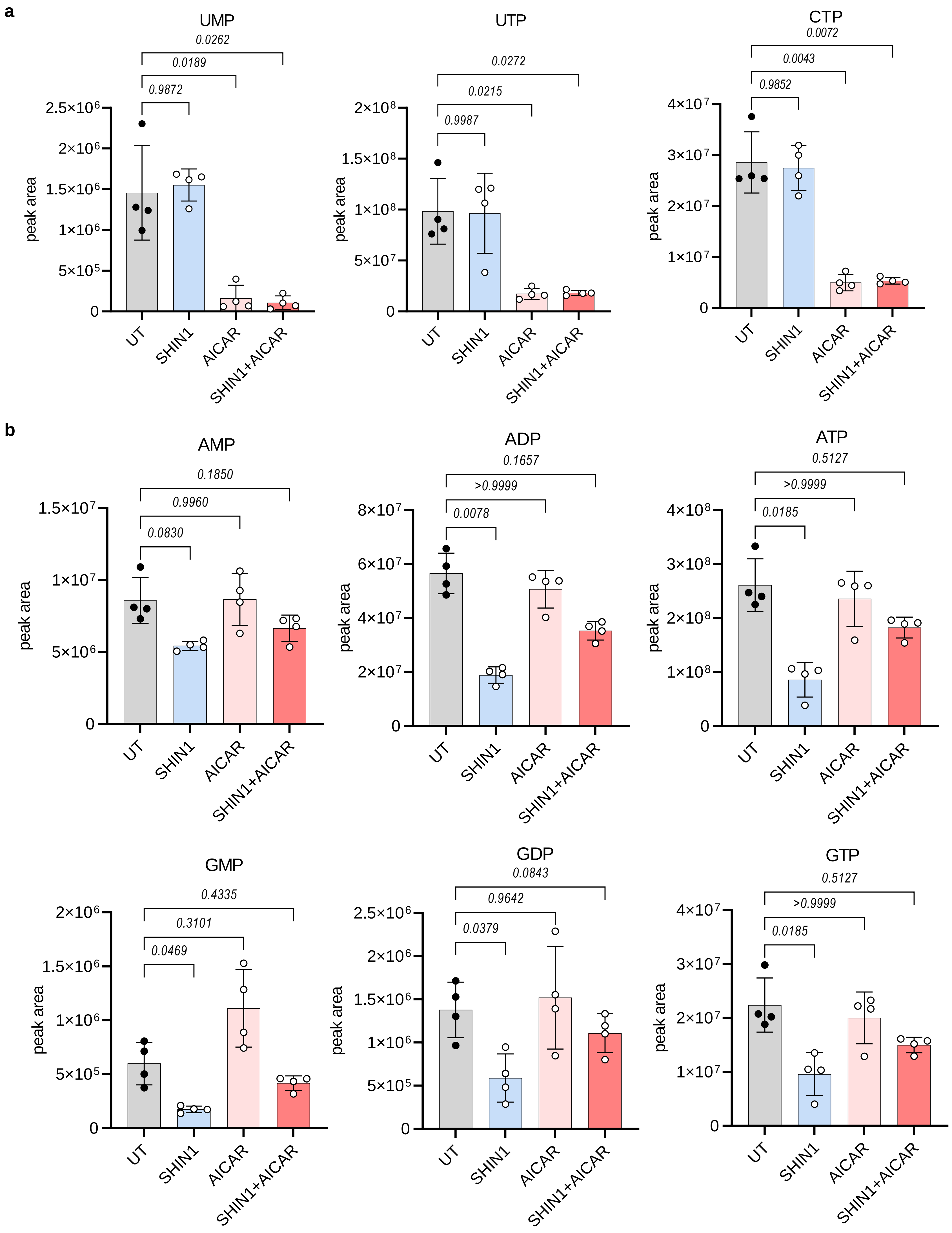

Supplementary Fig.6: AICAR exposure does not alter purine nucleotide levels. a,**b**, LC-MS measurement of intracellular pyrimidine nucleotides (UMP, UTP, CTP) (**a**) and purine nucleotides (AMP, ADP, ATP, GMP, GDP, GTP) (**b**) following 24 h incubation with 1 mM AICAR, 2.5 μM SHIN1, or a combination of both (n=4 independent cultures). Data are presented as the mean ± s.e.m. P-values were calculated using a repeated measure one-way ANOVA with Dunnett's multiple comparisons test (**a,b**). Source data are provided as a Source Data file.

Supplementary Figure 7:

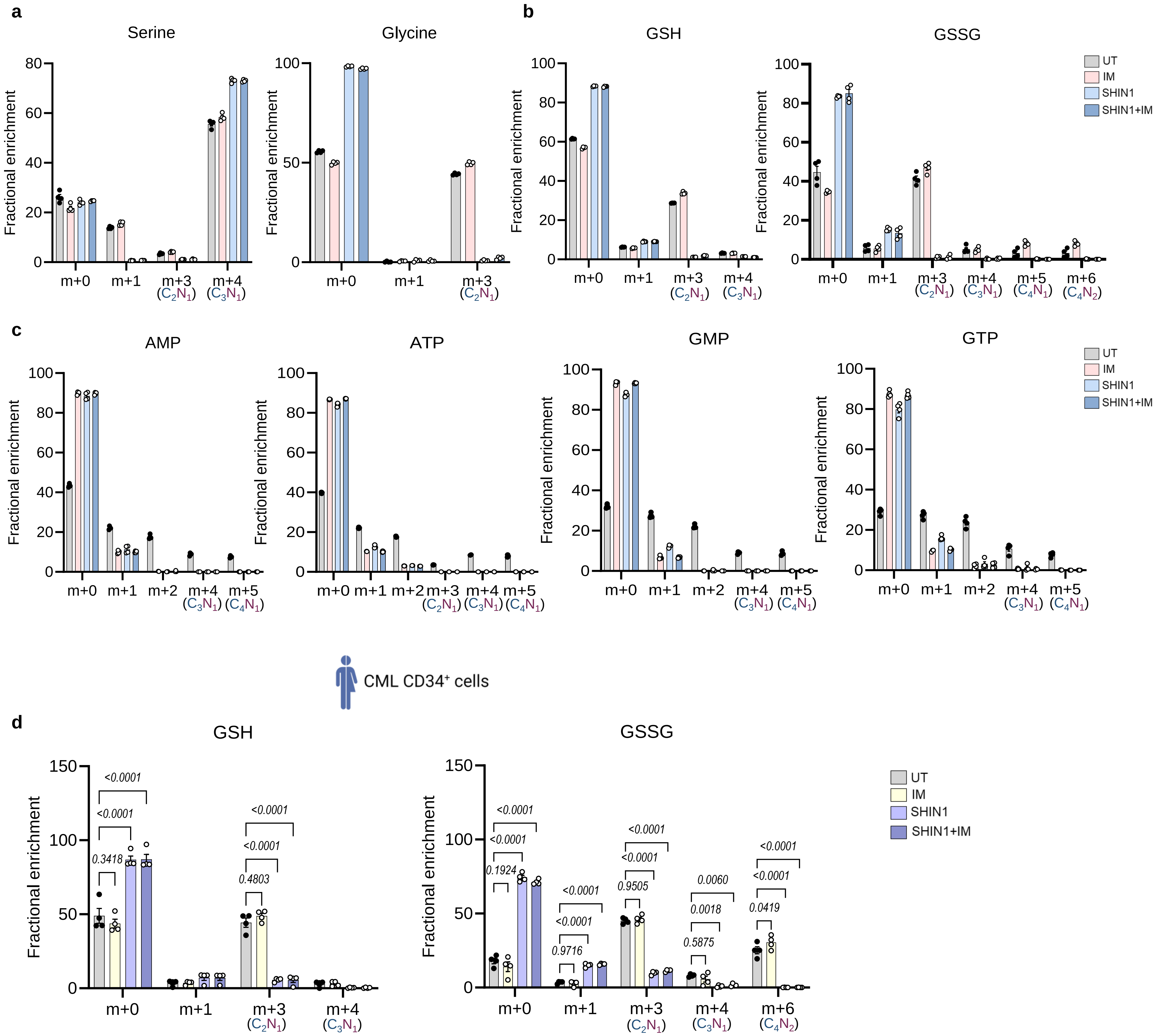

Supplementary Fig.7: Imatinib maintains serine contribution to glutathione. a-c, Relative mass isotopologue distribution in serine, glycine (**a**), GSH, GSSH (**b**) and purine nucleotides (**c**) from K562 cells treated for 24 h with 2 μM imatinib (IM), 2.5 μM SHIN1, or a combination of both in medium containing 140 μM $^{13}C_3{}^{15}N_1$-serine. (n=4 independent wells from individual experiment). **d**, Relative mass isotopologue distribution in GSG and GSSG from CML $CD34^+$ cells treated for 24 h with 2 μM imatinib, 2.5 μM SHIN1, or a combination of both in medium containing 140 μM $^{13}C_3{}^{15}N_1$-serine (n=4 patient samples). Each isotopologue is shown as a fraction of the sum of all possible isotopologues. Schematic created with Biorender.com (Agreement number SH26CFO8OE). Data are presented as the mean ± s.e.m. P-values were calculated using a repeated measure one-way ANOVA with Dunnett's multiple comparisons test (**d**). Source data are provided as a Source Data file.

Supplementary Figure 8:

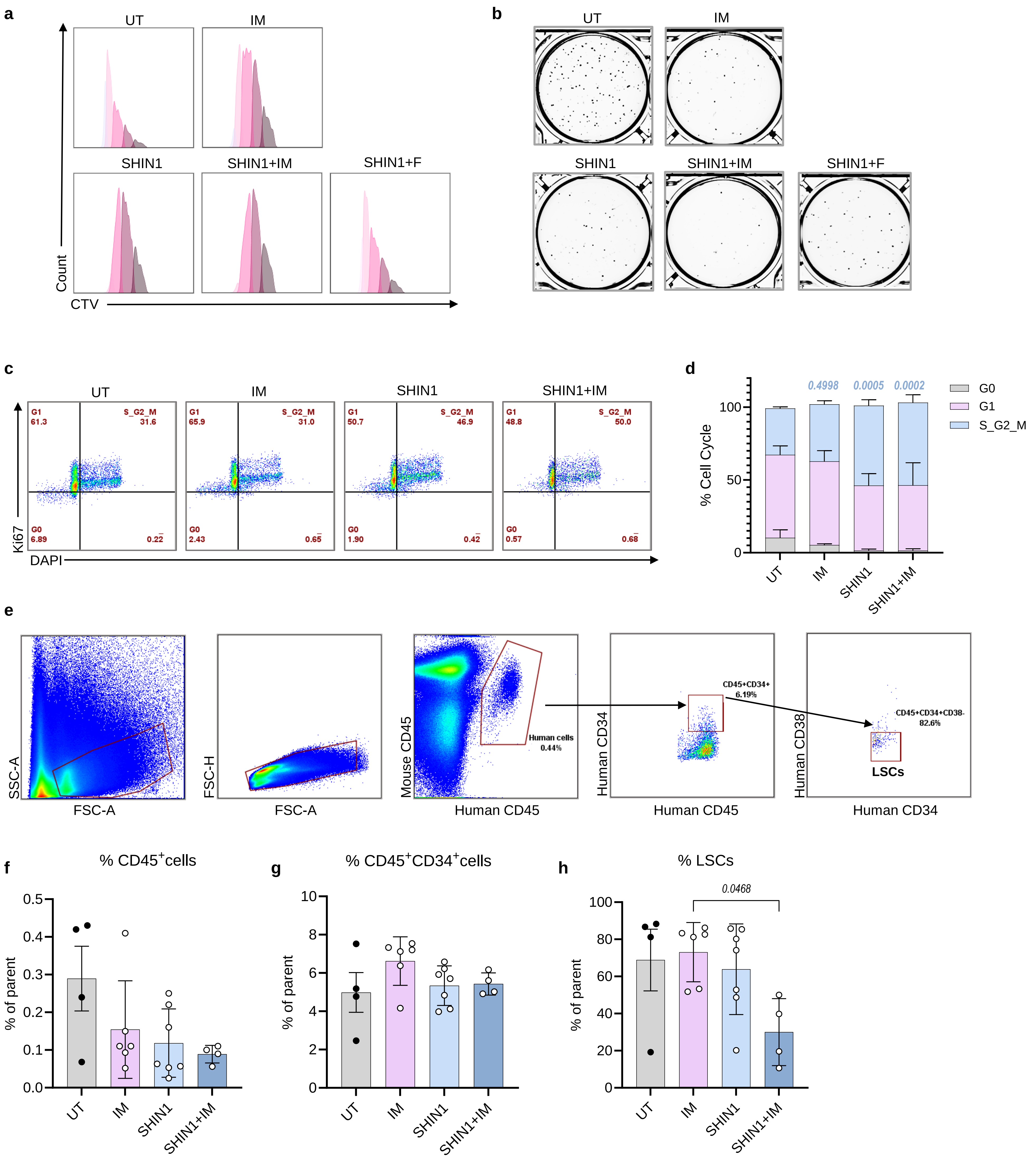

Supplementary Fig.8: **SHIN1 selectively targets LSCs in combination with standard CML therapy. a**, Representative flow cytometry histograms obtained from cell division tracking of CML CD34+ cells using CellTrace Violet (CTV) staining following 72 h of treatment with 2 μM imatinib (IM), 2.5 μM SHIN1 with or without the addition of 1mM formate, or a combination of imatinib and SHIN1. **b**, Representative images of colonies after exposure of CML CD34+ to treatment as in (**a**). **c**, Representative flow cytometry of Ki67 and DAPI levels in CML $CD34^+$ cells exposed to 2 μM imatinib, 2.5 μM SHIN1, or a combination of both for 48 h (n=4 patient samples). **d**, Percentage of cell cycle phases as depicted in (**c**). **e**, Gating strategy of flow cytometry analysis to measure engraftment of CML $CD34^+$ cells. **f-h**, Frequency of human $CD45^+$ (**d**), $CD34^+$ (**e**), and $CD34^+CD38^-$ (**f**) of CML cells from parent at experimental endpoint (n=4 mice for untreated group; n=6 mice for imatinib-treated group; n=7 mice for SHIN1-treated group, n=4 mice for combination group). Data are presented as the mean ± s.e.m. P-values were calculated with ordinary two-way ANOVA with Sidak's multiple comparisons test (**d**) and ordinary one-way ANOVA with Dunnett's multiple comparisons (**h**). P-values in (**d**) are derived from comparing the percentage of cells in the S_G2_M phase between UT, IM, SHIN1 and SHIN1 with IM. Source data are provided as a Source Data file.

Supplementary Table 1-Patient information		
ID	Other notes	Figures
CML 1	ELN failure Imatinib->nilotinib->dasatinib->SCT	Fig. 1c and Extended Data Fig.1d
CML 2	ELN failure: BCR-ABL 0.11% at 12 months, MMR by 18 months: not resistant	Fig. 1c and Extended Data Fig.1d
CML 3	Optimal response to imatinib (MR4)	Fig. 1c and Extended Data Fig.1d
CML 4	ELN warning on dasatinib	Fig. 3d, 6b
CML 5	Failed imatinib (compliance issues)	Fig. 3d, 6b
CML 6	Non-optimal response to imatinib 400 mg, therapy interrupted due to neutropenia intermittently -> dasatinib 100 mg daily: BCR-ABL PCR < 0.87% at 6 weeks	Fig. 3d, 5d,6a, 6b and Extended Data Fig. 7c, 8d
CML 7	Optimal response to imatinib (MR4)	Fig. 3d, 6b
CML 8	ELN warning	Fig. 5d, 6b and Extended Data Fig. 7c
CML 9	Treatment failure on imatinib-no CcyR by 12 months-> optimal response to dasatinib, now in DMR	Fig. 5d, 6b and Extended Data Fig. 7c
CML 10	Suboptimal response to imatinib 400 mg, increased to 600 mg, then reduced to 400 mg, optimal response: BCR-ABL 0.01%	Fig. 5d, 6b and Extended Data Fig. 7c
CML 11	Failed imatinib -> dasatinib, died	Fig. 6e-g and Extended Data Fig. 8f-h
CML 12	ELN warning in CP on imatinib	Extended data Fig. 8c,d
CML 13	ELN warning on nilotinib	Extended data Fig. 8d
CML 14	Imatinib daily 400 mg: BCR-ABL PCR > 1% at 12 months	Extended data Fig. 8d

Supplementary Table 1: information of patient samples (at diagnosis) used in this study.
-> refers to next treatment
ELN: European LeukaemiaNet (recommendations for the management of CML)
SCT: stem cell 5 transplantation
MMR: major molecular response
MR4: BCR-ABL ≤0.01% international scale (IS)
CcyR: complete cytogenetic remission, BCR-ABL ≤1% international scale (IS)
CP: refers to chronic phase

Supplementary Table 2 - sgRNA sequences	
Target	Guide sequence
AMPKα1	GAAGATCGGCCACTACATTC
AMPKα2	GAAGATCGGACACTACGTGC
ATG7	GAAGCTGAACGAGTATCGGC
SHMT2	GGACAGGCAGTGTCGTGGCC
TSC2	CACAAATCTGCCCTATCATC
ULK1	AGCAGATCGCGGGCGCCATG

Supplementary Table 2: Guide sequences used to generate knockout cell lines.

Supplementary Table 3 - Western Blot Antibodies		
Product	Manufacturer	Identifier
Primary antibodies		
β-tubulin	Cell Signaling Technology	Cat#2146 (1:2000)
ACC	Cell Signaling Technology	Cat#3662 (1:1000)
AMPKα	Cell Signaling Technology	Cat#2532 (1:1000)
ATG13	Cell Signaling Technology	Cat#13468, Clone E1Y9V (1:1000)
ATG7	Cell Signaling Technology	Cat#8558, Clone D12B11 (1:1000)
GAPDH	Cell Signaling Technology	Cat#5174, Clone D16H11 (1:5000)
HSP90	Proteintech	Cat#60318-1-Ig, Clone 3F11C1 (1:5000)
LC3B	Cell Signaling Technology	Cat#2775 (1:1000)
MTOR	Cell Signaling Technology	Cat#2983, Clone 7C10 (1:1000)
P62	BD Biosciences	Cat#610833, Clone 3 (1:1000)
phospho-ACC-Ser79	Cell Signaling Technology	Cat#3661 (1:1000)
phospho-AMPKα-Thr172	Cell Signaling Technology	Cat#2531 (1:1000)
phospho-ATG13-Ser355	Cell Signaling Technology	Cat#46329, Clone E4D3T (1:1000)
phospho-MTOR-Ser2448	Cell Signaling Technology	Cat#2971 (1:1000)
phospho-RPS6-Ser240/244	Cell Signaling Technology	Cat#5364, Clone D68F8 (1:1000)
phospho-S6K-Thr389	Cell Signaling Technology	Cat#9234, Clone 108D2 (1:1000)
phospho-ULK1-Ser555	Cell Signaling Technology	Cat#5869, Clone D1H4 (1:1000)
phospho-ULK1-Ser757	Cell Signaling Technology	Cat#6888 (1:1000)
RPS6	Cell Signaling Technology	Cat#2317, Clone 54D2 (1:1000)
S6K	Cell Signaling Technology	Cat#2708, Clone 4D7 (1:1000)
SHMT2	Cell Signaling Technology	Cat#33443, Clone E7F4Q (1:1000)
TSC2	Cell Signaling Technology	Cat#4308, Clone D93F12 (1:1000)
ULK1	Cell Signaling Technology	Cat#8054, Clone D8H5 (1:1000)
Secondary antibodies		
Anti-mouse IgG, HRP-linked	Cell Signaling Technology	Cat#7076 (1:3000)
Anti-rabbit IgG, HRP-linked	Cell Signaling Technology	Cat#7074 (1:3000)

Supplementary Table 3: List of western blot antibodies used.
